# Comparación de la métrica Sigma calculada con tres métodos de estimación del sesgo en 33 magnitudes químicas y 26 de inmunoensayo

**DOI:** 10.1515/almed-2023-0095

**Published:** 2023-08-28

**Authors:** Şerif Ercan

**Affiliations:** Departamento de Bioquímica Médica, Lüleburgaz State Hospital, Kırklareli, Türkiye

**Keywords:** Sesgo, química, control externo de la calidad, inmunoensayos, control de calidad interno, métrica Sigma

## Abstract

**Objetivos:**

Aunque la métrica Sigma se puede calcular mediante una sencilla ecuación, la diversidad de fuentes de las que se extraen los elementos de la ecuación pueden arrojar diferentes valores Sigma. El objetivo de este estudio era investigar la importancia de las distintas estrategias de estimación del sesgo para el cálculo de la métrica Sigma.

**Métodos:**

Se calculó la métrica Sigma de 33 magnitudes químicas y 26 magnitudes de inmunoensayo en un analizador Roche Cobas 6,000. El sesgo se calculó mediante tres métodos: a) calculando la media del sesgo mensual obtenida en los estudios de control de calidad externo (EQA, por sus siglas en inglés); 2) calculando los valores de sesgo mediante una ecuación de regresión a partir de datos obtenidos del EQA; y 3) calculando la media de los valores de sesgo mensual de los eventos de control de calidad internos (IQC, por sus siglas en inglés). Se realizó una métrica Sigma para cada uno de los dos niveles de muestras de IQC empleando tres métodos para calcular el sesgo. Los valores Sigma obtenidos se clasificaron en cinco categorías, en función de las reglas Sigma de Westgard, siendo ≥6, <6 y ≥5, <5 y ≥4, <4 y ≥3, y <3.

**Resultados:**

Al clasificar la métrica Sigma, calculada aplicando tres métodos de estimación del sesgo para cada magnitud, se observó que 16 magnitudes químicas en los niveles 1 y 2 de IQC fueron clasificadas en categorías Sigma diferentes por al menos uno de los métodos de estimación de la desviación. Del mismo modo, dependiendo del método de estimación del sesgo empleado, se clasificaba en diferentes categorías a 12 magnitudes de inmunoensayo con niveles 1 y 2 de IQC.

**Conclusiones:**

La métrica Sigma puede variar dependiendo del método empleado para calcular el sesgo, lo cual debe ser tenido en cuenta a la hora de evaluar el rendimiento analítico o programar eventos de IQC aplicando el método Seis Sigma.

## Introducción

Entre un 60 y un 70 % de los diagnósticos y tratamientos médicos se establecen en función de los datos obtenidos en el laboratorio clínico [1]. De este modo, con el objetivo de garantizar la seguridad del paciente, se deben observar todas las actividades que componen el proceso analítico total, que se desarrolla en tres fases: la fase preanalítica, la fase analítica, y la fase postanalítica, De manera rutinaria, se realizan procesos de control de la calidad, destinados a verificar la exactitud y precisión del proceso analítico. Así mismo, se puede evaluar la calidad analítica de un nuevo sistema, o de un sistema ya existente, aplicando otros métodos p.ej. Seis Sigma [2].

La metodología Seis Sigma se introdujo en la medicina de laboratorio en 2001 y sus implementaciones trascienden la aceptabilidad del método [3]. Con la métrica Sigma, los profesionales de laboratorio pueden definir las reglas de control de la calidad interno (IQC), así como el número de mediciones de control requeridas por serie, y aplicar un plan de IQC basado en los riesgos, determinando la frecuencia de los controles [2].

La metodología Seis Sigma combina los siguientes tres elementos: imprecisión, sesgo, y error total admisible (ETa) traduciéndolos a un único valor [2]. Existen múltiples fuentes de ETa objetivo en todo el mundo. En el documento de consenso de la 1^a^ Conferencia Estratégica de la Federación Europea de Química Clínica y Medicina de Laboratorio (EFLM), se recomiendan tres modelos para establecer las especificaciones de la calidad analítica (APS, por sus siglas en inglés): el primer modelo se basa en resultados clínicos, el segundo en variabilidad biológica, y el tercero en el estado del arte [4]. Para una magnitud dada, los valores de ETa pueden variar entre las distintas fuentes, lo que supone un problema para los laboratorios, a la hora de seleccionar las especificaciones de la calidad para realizar la métrica Sigma [5].

Tal como ocurre con los objetivos de ETa, existen diversos métodos para calcular la imprecisión y estimar la métrica Sigma. Para obtener datos de imprecisión, algunos estudios emplean el estudio de replicación, siguiendo un protocolo estandarizado en un periodo relativamente breve de tiempo [6], [7], [8], [9], [10]. En otros estudios, la imprecisión se calcula a partir de los datos obtenidos en el estudio de IQC a largo plazo, realizados de manera rutinaria [11], [12], [13], [14], [15].

Otro obstáculo a la hora de realizar la métrica Sigma es la estimación del sesgo, ya que este se puede realizar mediante diferentes métodos. Lo ideal a la hora de calcular el sesgo es comparar los resultados de un material de referencia o de las muestras del paciente que se vayan a analizar mediante un método de campo con los resultados de un método de referencia [16]. En estudios anteriores sobre las aplicaciones de Seis Sigma en el laboratorio clínico, el sesgo de una prueba concreta se suele obtener comparándolo con la media de grupo del programa de evaluación externa de la calidad (EQA). Con este método, los múltiples valores de sesgo obtenidos en los estudios de EQA se convierten en un valor de sesgo calculando la media [11], [12], [13, 15] o realizando un análisis de regresión [6, 17] para la métrica sigma. Otro método común para determinar el sesgo es calcular la diferencia entre los resultados de control observados y el valor (o media) del IQC objetivo [5, 10, 14, 15]. Así mismo, en algunos estudios, el sesgo se ha estimado realizando un análisis de regresión de los resultados obtenidos en estudios de comparación de métodos [7], [8], [9].

Estudios anteriores muestran que los valores Sigma dependen directamente de los objetivos de ETa obtenidos de diferentes fuentes [5, 15, 18]. Sin embargo, no existen datos suficientes sobre si los valores Sigma varían según el método empleado para estimar el sesgo.

El objetivo de este estudio fue comparar los valores Sigma de 33 magnitudes químicas y 26 magnitudes de inmunoensayo, que se determinaron utilizando tres métodos de cálculo del sesgo, incluyendo la media de los valores de desviación mensuales obtenidos de los estudios EQA a lo largo de un año, calculando los valores de sesgo mediante una ecuación de regresión a partir de datos de EQA, y calculando los valores medios de sesgo mensuales obtenidos en los estudios IQC según los valores objetivo de control asignados.

## Materiales y métodos

Este estudio observacional se realizó a partir de los datos de calidad EQA e IQC de 59 magnitudes, de las cuales 33 eran magnitudes químicas y 26 magnitudes de inmunoensayo, en el analizador Cobas 6,000 (Roche Diagnostics, Manheim, Alemania). En la Tabla 1 se enumeran las magnitudes evaluadas.

**Tabla 1:** Límites de error total admisible (ETa) y sus fuentes.

Magnitud	Fuente del ETa objetivo	TEa, %
Albúmina	CLIA	8,0
Fosfatasa alcalina (ALP)	Base de datos de VB de la SEQCML	12,0
Alanina aminotransferasa (ALT)	Base de datos de VB de la EFLM	16,1
Amilasa	Base de datos de VB de la EFLM	13,2
Antiestreptolisina O (ASO)	^a^	10,0
Aspartato aminotransferasa (AST)	Base de datos de VB de la EFLM	13,6
Bilirrubina, directa	RCPA	20,0
Bilirrubina, total	RCPA	12,0
Proteína C-reactiva (PCR)	RiliBAK	20,0
Calcio	RiliBAK	10,0
Cloro	RiliBAK	8,0
Colesterol HDL	NCEP	13,0
Colesterol LDL	NCEP	12,0
Colesterol, total	NCEP	8,9
Creatina quinasa	Base de datos de VB de la EFLM	22,6
Creatinina	Base de datos de VB de la EFLM	7,4
D-dímero	Base de datos de VB de la SEQCML	28,0
Gamma-glutamil transferasa (CGT)	Base de datos de VB de la EFLM	18,9
Glucosa	Base de datos de VB de la EFLM	6,5
Hemoglobina A1c	Grupo de Trabajo de la IFCC	6,9
Hierro	CLIA	15,0
Lactato deshidrogenasa (LDH)	Base de datos de VB de la EFLM	7,7
Lipasa	Base de datos de VB de la EFLM	14,2
Litio	CLIA	15,0
Magnesio	RCPA	8,0
Fósforo	Base de datos de VB de la EFLM	9,7
Potasio	RiliBAK	8,0
Proteínas totales	CLIA	8,0
Factor reumatoide	Base de datos de VB de la SEQCML	13,5
Sodio	RiliBAK	5,0
Triglicéridos	NCEP	15,0
Urea	Base de datos de VB de la EFLM	17,8
Ácido úrico	Base de datos de VB de la EFLM	12,8
25-OH Vitamina D	Base de datos de VB de la EFLM	12,4
Alfafetoproteína (AFP)	Base de datos de VB de la EFLM	17,6
Antígeno carbohidrato 125 (CA 125)	Base de datos de VB de la EFLM	13,9
Antígeno carbohidrato 15-3 (CA 15-3)	RiliBAK	24
Antígeno carbohidrato 19-9 (CA 19-9)	Base de datos de VB de la EFLM	17,9
Antígeno carcinoembriónico (CEA)	Base de datos de VB de la EFLM	20,5
Ferritin	Base de datos de VB de la SEQCML	16,9
Folato	Base de datos de VB de la EFLM	19,7
T3 libre	Base de datos de VB de la EFLM	6,5
T4 libre	Base de datos de VB de la EFLM	6,3
Hormona foliculoestimulante (FSH)	Base de datos de VB de la EFLM	21,2
Gonadotropina coriónica humana (hCG)	CLIA	18
Inmunoglobulina E (IgE)	CLIA	20
Insulina	Base de datos de VB de la EFLM	31,5
Hormona luteinizante (LH)	Base de datos de VB de la EFLM	28,4
Propéptido natriurético cerebral N-terminal (NT-ProBNP)	Base de datos de VB de la SEQCML	13
Estradiol	Base de datos de VB de la EFLM	17,3
Hormona paratiroidea	Base de datos de VB de la EFLM	20
Procalcitonina	^b^	20,3
Prolactina	Base de datos de VB de la EFLM	37,4
PSA, libre	Base de datos de VB de la EFLM	17,5
PSA, Total	Base de datos de VB de la EFLM	16,2
Testosterona	Base de datos de VB de la EFLM	16,5
Troponina T	Base de datos de VB de la EFLM	17,6
Hormona estimulante de la tiroides (TSH)	Base de datos de VB de la EFLM	24,6
Vitamina B12	CLIA	20

Base de datos de VB de la EFLM, Base de atos de variación biológica de la Federación Europea de Química Clínica y Medicina de Laboratorio; CLIA, Clinical Laboratory Improvement Amendments [25]; RCPA, The Royal College of Pathologists of Australasia [26]; RiliBAK, German Medical Association on Quality Assurance in Medical Laboratory Examinations [27]; Base de datos de VB de la SEQCML, base de datos de variabilidad biológica de la Comisión de Calidad Analítica de la Sociedad Española de Medicina de Laboratorio [24]; NCEP, Programa Nacional de Educación sobre el Colesterol [19], [20], [21]; Grupo de Trabajo de la IFCC, Grupo de Trabajo de la Federación Internacional de Química Clínica y Medicina de Laboratorio para la implementación de la estandarización de la HbA_1c_ [22]. ^a^El ETa para ASO se eligió de manera arbitraria, ante la ausencia de datos. ^b^Referencia [28].

La métrica Sigma se realizó empleando el ETa, el sesgo y la precisión (expresada como coeficiente de variación, %CV) aplicando la siguiente fórmula: Sigma=[(%ETa)-|Sesgo|]/%CV.

### Error total admisible (ETa)

Existen múltiples fuentes para obtener el ETa, aunque ninguna de ellas abarca la totalidad de las magnitudes evaluadas en este estudio.

A la hora de seleccionar las especificaciones de la calidad analítica, se siguió el modelo jerárquico propuesto en Estocolmo en 1999 y en Milán en 2014 [4]. En dicho modelo, la fuente principal a la hora de seleccionar las especificaciones de calidad es el resultado clínico, aunque desafortunadamente, solo se dispone de los resultados clínicos de unas pocas magnitudes. Se seleccionaron objetivos de ETa para triglicéridos [19], colesterol total [20], colesterol de lipoproteínas de alta densidad [20], colesterol de lipoproteínas de baja densidad [21], y hemoglobina glicosilada (HbA_1c_) [22] según el resultado clínico.

La segunda opción fue el ETa deseable, en función de la variación biológica extraida de la Base de Datos de Variación Biológica de la EFLM [23]. Si algún dato no estaba disponible en esta base de datos, se consultó la base de datos de variación biológica creada por la Comisión de Calidad Analítica de la Sociedad Española de Medicina de Laboratorio, que se lleva actualizando cada dos años desde 2014 [24]. Si los objetivos de variación biológica no eran prácticos (demasiado estrictos) o resultaban insuficientes (demasiado permisivos) para alguna magnitud, se utilizaron los objetivos del modelo CLIA [25], del Colegio Real de Patólogos de Australasia (RCPA) [26], y de las guías alemanas Rili-BAEK de la Asocación Médica Alemana de Control de la Calidad en los Análisis Clínicos [27].

Finalmente, ninguna de las fuentes proporcionaba datos de ETa para la procalcitonina y la anti-estreptolisina O (ASO). El objetivo deETa para la procalcitonina se extrajo de un artículo sobre variación biológica [28], mientras que el objetivo de ETa para ASO se fijó en 10 %.

En la Tabla 1 se muestra la fuente principal de ETa.

### Sesgo

Para calcular el sesgo, se extrajeron datos de EQA de los programas de EQA de RIQAS (bioquímica clínica mensual, proteínas específicas, hemoglobina glicosilada, lípidos, inmunoanálisis mensual, control de marcadores cardíacos, control de especialidad de inmunoanálisis) a lo largo de un año, entre el 1 de marzo de 2019 y el 29 de febrero de 2020.

El sesgo de cada magnitud se estimó mediante tres métodos. En el primer método, se calculó la diferencia entre el resultado del laboratorio y el valor medio del grupo par, en cada una de las doce evaluaciones de EQA. A continuación, se calculó el sesgo medio, estimando la raíz cuadrada media de los valores de sesgo individuales.

En el segundo método, se realizó un análisis de regresión Passing-Bablok [29]empleando los resultados del laboratorio y los valores medios del grupo par obtenidos en las doce evaluaciones de EQA. La desviación se calculó aplicando la ecuación de regresión y=b+ax, donde a era la pendiente, b era ordenada en el origen y x era el valor que contenía la concentración dentro de la media de la muestra de IQC. Con este método, la desviación de cada analito se calculó por separado, distinguiendo entre el nivel 1 y el nivel 2.

Cuando se utilizó el método de cálculo del sesgo basado en los resultados de EQA, si el índice de desviación estándar de un ensayo era >3 o <−3 en un evento de EQA, el valor de desviación correspondiente se excluyó de la métrica Sigma.

En el último método, la diferencia entre el resultado del laboratorio y el valor objetivo de la muestra de IQC obtenido a partir de los “*inserts”* del control, se calculó de forma mensual durante un año, y posteriormente se calculó el sesgo medio a partir de la raíz cuadrada media de los valores mensuales de desviación. Las muestras de IQC fueron suministradas por el fabricante del reactivo (Roche Diagnostics, Manheim, Alemania). Al igual que en el segundo método, el sesgo de cada magnitud se calculó por separado, distinguiendo entre el nivel 1 y 2 de IQC.

### Precisión

La precisión se determinó calculando el %CV de los datos de IQC. Los datos de IQC y de EQA se recogieron en el mismo periodo de tiempo.

Los valores de %CV se calcularon mensualmente, para posteriormente calcular la precisión media. Los valores de precisión de las dos muestras de IQC se estimaron por separado.

### Clasificación de la métrica Sigma

Las métricas Sigma calculadas empleando valores de sesgo obtenidos mediante los diferentes métodos de estimación se definieron de la siguiente manera:

1. Si un valor Sigma se había estimado a partir de valores de sesgo basados en la media de varios valores de sesgo extraidos de las encuestas de EQA, se le llamó “Sigma medio de EQA”.
2. Si el valor de Sigma se había calculado empleando valores de sesgo obtenidos mediante el análisis de regresión de los resultados de EQA, se le llamó “Sigma de Regresión de EQA”.
3. Si un valor Sigma se había estimado a partir de valores de sesgo basados en la media de los valores de sesgo obtenidos en las encuestas de IQC, se le llamó “Sigma de IQC”.

Los valores Sigma resultantes se clasificaron en función del diagrama de las “reglas Sigma de Westgard” [30] de la siguiente manera: categoría 1 (<3 de Sigma), 2 (≥3 y <4), 3 (≥4 y <5), 4 (≥5 y <6), y 5 (≥6). A continuación, el número de magnitudes químicas y de inmunoanálisis que se incluyeron en cada categoría Sigma se determinó según el método de empleado para calcular el sesgo.

Los análisis de datos se realizaron con el programa MedCalc Statistical versión 19.1 (MedCalc Software Ltd, Ostend, Belgium) y Excel Office 2019 (Microsoft, USA).

## Resultados y discusión

En las Tablas 2 y 3 se muestran los valores Sigma calculados con los diferentes métodos de determinación del sesgo para los niveles 1 y 2 de IQC, respectivamente.

**Tabla 2:** Métrica Sigma calculada con tres métodos de cálculo en el nivel 1 de IQC.

Magnitud	%CV (Nivel 1)	Valores de sesgo de la media de los resultados de los programas de EQA, %	Valores de sesgo del análisis de regresión de los resultados de EQA	Valores de sesgon de los resultados de IQC	Valores “Sigma media de EQA”	Valores “Sigma de regresión de EQA”	Valores “Sigma de IQC”	Categoría Sigma basada en los valores “Sigma medio de EQA”^a^	Categoría Sigma basada en los valores “Sigma de regresión de EQA”^a^	Categoría Sigma basada en los valores “Sigma de IQC”^a^
Albúmina	2,58	3,99	1,49	1,37	1,6	2,5	2,6	1	1	1
ALP	2,52	2,15	0,76	2,96	3,9	4,5	3,6	2	3	2
ALT	1,82	1,87	0,76	2,12	7,8	8,4	7,7	5	5	5
Amilasa	1,38	2,49	1,36	1,16	7,7	8,6	8,7	5	5	5
Antiestreptolisina O	3,10	3,88	2,93	3,47	2,0	2,3	2,1	1	1	1
AST	2,02	4,08	2,65	1,06	4,7	5,4	6,2	3	4	5
Bilirrubina, directa	2,26	1,78	0,99	2,17	8,1	8,4	7,9	5	5	5
Bilirrubina, total	2,18	1,88	1,67	1,79	4,6	4,7	4,7	3	3	3
Proteína C-reactiva	2,54	2,23	7,54	3,17	7,0	4,9	6,6	5	3	5
Calcio	1,41	2,13	0,88	1,06	5,6	6,5	6,3	4	5	5
Cloro	2,33	2,00	1,46	2,18	2,6	2,8	2,5	1	1	1
Colesterol, HDL	2,28	3,52	11,76	2,93	4,2	0,5	4,4	3	1	3
Colesterol, LDL	2,73	3,15	0,33	3,70	3,2	4,3	3,0	2	3	2
Colesterol, total	1,95	2,27	0,91	2,90	3,4	4,1	3,1	2	3	2
Creatina quinasa	1,49	1,86	2,52	0,63	13,9	13,5	14,8	5	5	5
Creatinina	3,22	2,83	1,09	2,39	1,4	2,0	1,6	1	1	1
D-dímero	2,64	5,58	6,57	3,68	8,5	8,1	9,2	5	5	5
GGT	1,58	3,35	2,04	1,88	9,8	10,7	10,8	5	5	5
Glucosa	1,50	1,51	0,02	1,85	3,3	4,3	3,1	2	3	2
Hemoglobina a1c	1,66	1,85	1,00	0,88	3,0	3,6	3,6	2	2	2
Hierro	2,28	1,97	0,73	1,58	5,7	6,3	5,9	4	5	4
LDH	1,51	1,68	0,41	2,58	4,0	4,8	3,4	3	3	2
Lipasa	2,05	2,68	1,78	1,55	5,6	6,1	6,2	4	5	5
Litio	3,12	3,18	1,70	3,68	3,8	4,3	3,6	2	3	2
Magnesio	1,88	2,10	0,87	1,13	3,1	3,8	3,6	2	2	2
Fósforo	1,70	1,84	0,04	1,97	4,6	5,7	4,6	3	4	3
Potasio	1,79	1,37	0,01	0,65	3,7	4,5	4,1	2	3	3
Proteínas totales	1,46	2,79	1,76	1,79	3,6	4,3	4,3	2	3	3
Factor reumatoide	2,58	1,84	1,88	2,24	4,5	4,5	4,4	3	3	3
Sodio	1,57	1,46	1,21	0,60	2,3	2,4	2,8	1	1	1
Triglicéridos	1,66	2,68	1,36	1,58	7,4	8,2	8,1	5	5	5
Urea	1,91	2,64	0,85	1,76	7,9	8,9	8,4	5	5	5
Ácido úrico	1,79	2,25	1,29	1,30	5,9	6,4	6,4	4	5	5
25-OH Vitamina D	9,47	11,89	14,64	5,26	0,1	<0	0,2	1	1	1
AFP	2,75	6,35	2,34	2,21	4,1	5,5	5,6	3	4	4
CA 125	2,31	8,17	7,85	4,59	2,5	2,6	4,0	1	1	3
CA 15-3	4,01	6,13	1,20	5,10	4,5	5,7	4,7	3	4	3
CA 19-9	2,75	7,24	1,41	3,82	3,9	6,0	5,1	2	5	4
CEA	2,35	4,32	1,51	1,74	6,9	8,1	8,0	5	5	5
Ferritina	2,97	6,75	10,22	3,25	3,4	2,3	4,6	2	1	3
Folato	7,37	8,45	5,41	6,94	1,5	1,9	1,7	1	1	1
T3 libre	2,89	4,26	2,91	2,63	0,8	1,2	1,3	1	1	1
T4 libre	3,63	2,81	3,95	2,36	1,0	0,6	1,1	1	1	1
FSH	2,23	6,31	5,23	2,40	6,7	7,2	8,4	5	5	5
hCG	3,33	6,97	7,48	3,90	3,3	3,2	4,2	2	2	3
IgE	2,25	6,27	6,38	3,35	6,1	6,1	7,4	5	5	5
Insulina	2,17	4,13	4,69	2,07	12,6	12,4	13,6	5	5	5
LH	2,09	3,79	2,87	2,70	11,8	12,2	12,3	5	5	5
NT-ProBNP	3,28	3,74	1,50	2,68	2,8	3,5	3,1	1	2	2
Estradiol	3,89	4,24	8,40	6,32	3,4	2,3	2,8	2	1	1
PTH	1,83	8,21	2,93	6,76	6,4	9,3	7,2	5	5	5
Procalcitonina	2,49	3,45	1,26	3,02	6,8	7,6	6,9	5	5	5
Prolactina	2,48	4,54	4,51	1,51	13,2	13,4	14,4	5	5	5
PSA, libre	2,50	3,73	4,87	2,40	5,5	5,1	6,0	4	4	5
PSA, Total	2,08	5,89	8,62	2,16	5,0	3,6	6,8	4	2	5
Testosterona	1,98	4,40	2,46	4,26	6,1	7,1	6,2	5	5	5
Troponina Ths	2,95	6,82	2,69	2,93	3,7	5,1	5,0	2	4	4
TSH	1,91	1,92	0,70	2,99	11,9	12,5	11,3	5	5	5
Vitamina B12	4,38	4,53	3,70	2,76	4,7	4,9	5,1	3	3	4

^a^Los valores Sigma resultantes se clasificaron en función del diagrama [de las “reglas Sigma de Westgard” ]30≥ de la siguiente manera: categoría 1 (<3 de sigma), 2 (≥3 y <4), 3 (≥4 y <5), 4 (≥5 y <6), y 5 (6).

**Tabla 3:** Métrica Sigma calculada con tres métodos de estimación en el nivel 2 de IQC.

Magnitud	%CV (Nivel 2)	Valores de sesgo de la media de los resultados de los programas de EQA, %	Valores de sesgo del análisis de regresión de los resultados de EQA	Valores de sesgo de los resultados de IQC	Valores “Sigma media de EQA”	Valores “Sigma de regresión de EQA”	Valores “Sigma de IQC”	Categoría Sigma basada en los valores “Sigma medio de EQA”^a^	Categoría Sigma basada en los valores “Sigma de regresión de EQA”^a^	Categoría Sigma basada en los valores “Sigma de IQC”^a^
Albúmina	1,61	3,99	1,68	1,25	2,5	3,9	4,2	1	2	3
ALP	2,10	2,15	0,53	6,13	4,7	5,5	2,8	3	4	1
ALT	1,46	1,87	1,16	1,57	9,7	10,2	9,9	5	5	5
Amilasa	1,40	2,49	0,22	1,73	7,6	9,2	8,2	5	5	5
Antiestreptolisina O	2,83	3,88	0,65	2,72	2,2	3,3	2,6	1	2	1
AST	1,60	4,08	3,19	1,98	6,0	6,5	7,3	5	5	5
Bilirrubina, directa	1,88	1,78	0,75	1,19	9,7	10,2	10,0	5	5	5
Bilirrubina, total	1,84	1,88	0,17	1,83	5,5	6,4	5,5	4	5	4
Proteína C-reactiva	1,89	2,23	0,68	4,73	9,4	10,2	8,1	5	5	5
Calcio	1,38	2,13	1,49	1,20	5,7	6,2	6,4	4	5	5
Cloro	1,79	2,00	0,00	1,86	3,4	4,5	3,4	2	3	2
Colesterol, HDL	1,95	3,52	1,46	4,11	4,9	5,9	4,6	3	4	3
Colesterol, LDL	2,43	3,15	0,08	2,87	3,6	4,9	3,8	2	3	2
Colesterol, total	1,46	2,27	0,94	1,08	4,6	5,5	5,4	3	4	4
Creatina quinasa	1,44	1,86	0,68	0,78	14,4	15,2	15,1	5	5	5
Creatinina	2,40	2,83	3,40	1,78	1,9	1,7	2,3	1	1	1
D-dímero	1,31	5,58	8,70	1,56	17,1	14,7	20,2	5	5	5
GGT	1,32	3,35	1,63	1,44	11,7	13,0	13,2	5	5	5
Glucosa	1,35	1,51	0,62	0,51	3,7	4,4	4,4	2	3	3
Hemoglobina A1c	1,47	1,85	0,66	0,79	3,4	4,3	4,2	2	3	3
Hierro	2,00	1,97	1,81	0,68	6,5	6,6	7,2	5	5	5
LDH	1,41	1,68	0,28	2,11	4,3	5,2	4,0	3	4	3
Lipasa	1,87	2,68	1,96	2,74	6,2	6,6	6,1	5	5	5
Litio	1,98	3,18	1,12	1,33	6,0	7,0	6,9	5	5	5
Magnesio	1,73	2,10	1,79	0,88	3,4	3,6	4,1	2	2	3
Fósforo	1,43	1,84	0,80	2,05	5,5	6,2	5,4	4	5	4
Potasio	1,49	1,37	0,99	0,66	4,5	4,7	4,9	3	3	3
Proteínas totales	1,29	2,79	2,16	1,34	4,0	4,5	5,2	3	3	4
Factor reumatoide	1,77	1,84	0,24	1,33	6,6	7,5	6,9	5	5	5
Sodio	1,55	1,46	0,28	0,68	2,3	3,0	2,8	1	2	1
Triglicéridos	1,38	2,68	1,37	1,84	8,9	9,9	9,5	5	5	5
Urea	1,78	2,64	0,63	1,86	8,5	9,6	8,9	5	5	5
Ácido úrico	1,63	2,25	0,02	1,48	6,5	7,8	6,9	5	5	5
25-OH Vitamina D	3,58	4,53	4,66	2,18	0,1	0,3	1,5	1	1	1
AFP	2,78	6,35	7,78	3,61	4,0	3,5	5,0	3	2	4
CA 125	2,25	8,17	8,70	3,03	2,5	2,3	4,8	1	1	3
CA 15-3	3,36	6,13	6,52	3,02	5,3	5,2	6,2	4	4	5
CA 19-9	2,42	7,24	8,62	3,81	4,4	3,8	5,8	3	2	4
CEA	2,09	4,32	4,94	1,88	7,7	7,4	8,9	5	5	5
Ferritina	2,26	6,75	9,18	2,72	4,5	3,4	6,3	3	2	5
Folato	5,67	8,45	5,79	6,03	2,0	2,5	2,4	1	1	1
T3 libre	2,11	4,26	3,72	2,89	1,1	1,3	1,7	1	1	1
T4 libre	3,69	2,81	1,09	2,72	0,9	1,4	1,0	1	1	1
FSH	2,09	6,31	7,60	1,79	5,6	5,0	7,8	4	4	5
hCG	3,06	6,97	5,39	3,50	3,6	4,1	4,7	2	3	3
IgE	1,99	6,27	7,01	3,30	6,9	6,5	8,4	5	5	5
Insulina	2,05	4,13	4,99	2,07	13,3	12,9	14,3	5	5	5
LH	1,94	3,79	4,21	1,60	12,7	12,5	13,8	5	5	5
NT-ProBNP	2,42	3,74	2,63	2,69	3,8	4,3	4,3	2	3	3
Estradiol	2,51	4,24	1,33	8,44	5,2	6,4	3,5	4	5	2
PTH	1,75	8,21	4,40	5,74	6,7	8,9	8,1	5	5	5
Procalcitonina	2,30	3,45	4,10	3,19	7,3	7,0	7,4	5	5	5
Prolactina	2,38	4,54	4,11	1,22	13,8	13,8	15,2	5	5	5
PSA, libre	2,48	3,73	3,69	2,56	5,6	5,6	6,0	4	4	5
PSA, Total	2,05	5,89	4,72	1,83	5,0	5,6	7,0	4	4	5
Testosterona	2,68	4,38	2,20	5,90	4,5	5,3	4,0	3	4	3
Troponina Ths	1,94	1,92	2,40	2,35	5,1	5,4	7,3	4	4	5
TSH	5,61	11,89	10,47	3,77	11,7	11,5	11,5	5	5	5
Vitamina B12	2,12	6,82	6,15	2,08	6,2	6,5	8,4	5	5	5

^a^Los valores Sigma resultantes se clasificaron en función del diagrama [de las “reglas Sigma de Westgard” ]30≥ de la siguiente manera: categoría 1 (<3 de sigma), 2 (≥3 y <4), 3 (≥4 y <5), 4 (≥5 y <6), y 5 (6).

En el material suplementario se pueden encontrar los datos empleados para calcular los valores de sesgo. Los valores de sesgo mensuales obtenidos a partir de los resultados de EQA se presentan por meses en la Tabla Suplementaria 1. No se excluyó ningún valor de sesgo debido al índice de desviación estándar. Los valores mensuales de sesgo calculados a partir de los resultados de IQC para los niveles de control 1 y 2 se muestran en las Tablas Suplementarias 2 y 3, respectivamente. Además, la Tabla Suplementaria 4 muestra la ecuación de regresión derivada del análisis de regresión Passing-Bablok para cada magnitud, así como los valores objetivo empleados para calcular el sesgo.

En el 64 % de las magnitudes químicas en el nivel 1 de IQC y el 73 % de las magnitudes en el nivel 2, los valores “Sigma de regresión de EQA” fueron superiores a los valores “Sigma medio de EQA” en al menos 0,5 puntos. Del mismo modo, en el 42 % de las mangnitudes químicas en el nivel 1 de IQC y en el 45 % de las magnitudes en el nivel 2, los valores “Sigma de IQC” fueron superiores a los valores “Sigma medio de EQA”. Además, al compararlos con los valores “Sigma de IQC”, los valores “Sigma de regresión de EQA” fueron superiores en 9 magnitudes químicas, mientras que los valores “Sigma IQC” fueron superiores en 5 magnitudes químicas. Estos hallazgos demuestran que la estimación del sesgo basado en el análisis de regresión de los resultados de la EQA podrían arrojar resultados Sigma superiores para las magnitudes químicas.

Al contrario de lo que ocurre en las magnitudes químicas, para las magnitudes de inmunoanálisis, los valores Sigma estimados empleando valores de sesgo basados en los resultados de IQC fueron superiores a los obtenidos con otros métodos. En el 65 % de las magnitudes de inmunoanálisis en el nivel 1 de IQC y en el 69 % en el nivel 2, los valores “Sigma de IQC” fueron superiores a los valores “Sigma medio de EQA” en al menos 0,5 puntos. Del mismo modo, en el 42 % de las magnitudes de inmunoanálisis en el nivel 1 de IQC y en el 62 % de las magnitudes en el nivel 2, los valores “Sigma de IQC” fueron superiores a los valores “Sigma de regresión de EQA”. Así mismo, se observó una diferencia de al menos 0,5 puntos entre los valores “Sigma medio de EQA” en 13 ensayos en el nivel 1 de IQC y en 9 magnitudes en el nivel 2 de IQC.

Esta diferencia llevó a clasificar a algunas magnitudes en diferentes categorías Sigma. Un total de 23 magnitudes (15 químicas y 8 de inmunoanálisis) en el nivel 1 de IQC y 21 magnitudes (14 químicas y 7 de inmunoanálisis) en el nivel 2 fueron clasificadas en categorías Sigma diferentes, según se considerara el valor “Sigma medio de EQA” o “Sigma de regresión de EQA”. Además, en 18 magnitudes (7 químicas, 11 de inmunoanálisis) en el nivel 1 de IQC y 20 (8 químicas, 12 de inmunoanálisis) en el nivel 2, se asignaron categorías Sigma diferentes, dependiendo de si se aplicaban los valores “Sigma medio de EQA” o el valor “Sigma de IQC”. Del mismo modo, cuando el sesgo se calculó mediante el análisis de regresión de los valores de sesgo obtenidos en los estudios EQA en lugar de IQC, se clasificó en categorías Sigma diferentes a 19 magnitudes (11 químicas, 8 de inmunoanálisis) en el nivel 1 de IQC y 23 (12 químicas, 11 de inmunoanálisis) en el nivel 2. En la mayoría de las magnitudes con un valor Sigma superior a 6, las categorías Sigma asignadas no variaron, independientemente del método empleado para calcular la desviación. Esto se debe a que los valores Sigma iguales o superiores a 6 se clasificaban en la misma categoría, por lo que cuanto mayor fuera el valor Sigma, mayor sería la diferencia necesaria para producir un cambio en dicha categoría Sigma. Por ejemplo, aunque hubo una diferencia de 2,2 entre el valor “Sigma medio de EQA” y el valor “Sigma de regresión de EQA” para PTH en el nivel 2 de IQC, los dos fueron asignados a la categoría Sigma 5.

En el presente estudio, los valores Sigma se separaron en categorías siguiendo el diagrama de las Reglas Sigma de Westgard [30]. Por lo tanto, la diferencia observada en la categoría Sigma según el método de estimación del sesgo para un ensayo puede alterar las reglas del control de calidad, el número de mediciones de control requeridas por serie y la frecuencia de medición de las muestras de control. Si tomamos como ejemplo AST, la calidad de “Sigma IQC” en el nivel 1 de IQC solo requiere una única regla de control, 1_3s_, con 2 mediciones de control en cada serie en cada nivel de control, mientras que la calidad “Sigma media de EQA” requiere 4 reglas, 1_3s_/2_2s_/R_4s_/4_1s_, con 4 mediciones de control en cada serie o 2 mediciones de control en cada una de las 2 series.

Además, las variaciones observadas en las categorías Sigma habrán modificado los resultados sobre la calidad de un sistema analítico existente en el laboratorio. Al evaluar la calidad analítica de las magnitudes químicas, considerando un valor Sigma <3 como inaceptable, se clasificó la albúmina, creatinina, sodio, cloro y ASO en esta categoría en el nivel 1 de IQC, independientemente del método empleado para calcular el sesgo. Además de estas magnitudes, el valor “Sigma de regresión de EQA” para el colesterol HDL fue <3. En el nivel 2 de IQC, la creatinina, el sodio y el ASO mostraron una calidad Sigma <3, considerando tanto los valores “Sigma de IQC” como los valores “Sigma medio de EQA”. Además, ALP mostró un valor “Sigma de IQC” <3.

En los niveles 1 y 2 de IQC, FT3, FT4, el ácido fólico y la 25-OH vitamina D mostraron una calidad Sigma <3, independientemente del método de estimación del sesgo empleado. Además de estas magnitudes, el valor “Sigma medio de EQA” y el valor “Sigma de regresión de EQA” para CA 125 fue <3, tanto en el nivel 1 como en el nivel 2 de IQC. Por otro lado, en el nivel 1 de IQC, el estradiol mostró una calidad Sigma <3, teniendo en cuenta el valor “Sigma de regresión de EQA” o el valor “Sigma de IQC”. Lo mismo se observó en el NT-ProBNP, con un valor “Sigma medio de EQA” <3, y la ferritina, con un valor “Sigma de regresión de EQA” <3.

Cada uno de los métodos empleados en este estudio para la estimación del sesgo tienen sus ventajas y sus inconvenientes. Lo ideal sería que las organizaciones de EQA emplearan materiales de control conmutables, con valores asignados mediante métodos de referencia para verificar la veracidad de los resultados analíticos y su impacto en las muestras de los pacientes [31]. Sin embargo, varios proveedores de EQA emplean materiales de control conmutables o no conmutables, pero sin valores asignados mediante métodos de referencia [31]. Por otro lado, no existen materiales o métodos de referencia para varias de las magnitudes que se emplean con frecuencia en medicina de laboratorio. Los valores objetivo de EQA empleados en el presente estudio se establecieron en función de los valores medios obtenidos en grupos par (laboratorios que utilicen el mismo método, analizador y reactivo) En dicho caso, la incertidumbre del valor medio del grupo par, que depende de la imprecisión de los datos empleados y del número de laboratorios incluidos en el grupo comparable influye en el sesgo observado. Aunque existe una relación directa entre la incertidumbre y el tamaño de la muestra del grupo par, el número de participantes requeridos en un grupo par no se ha estandarizado aún. En un estudio previo [17], se consideró que un grupo par con al menos cinco participantes era suficiente para calcular el sesgo. En este estudio, el número de laboratorios en el grupo par fue relativamente alto, con al menos 100 participantes en los inmunoanálisis y 30 en los análisis químicos.

Para calcular el sesgo a partir de los resultados de EQA, otra limitación fue que el sesgo calculado en cada evento de EQA se basaba en una única medición. Así, el sesgo calculado con este método es susceptible de errores aleatorios, especialmente cuando se emplean valores de sesgo basados en pocos estudios de EQA. Calcular el sesgo durante un periodo más prolongado podría ayudar a reducir el efecto de los posibles errores aleatorios. En un estudio reciente [15], los autores recomendaron que los laboratorios calcularan la métrica Sigma empleando datos correspondientes, al menos, a seis meses. Por otro lado, al utilizar el análisis de regresión para calcular el sesgo a partir de resultados de EQA, el tamaño muestral se convierte en un aspecto muy complejo. El número de pares de datos recomendado para el análisis de regresión de Passing-Bablok es de 30 [32]. Sin embargo, no resulta fácil alcanzar dicho tamaño muestral a través de las evalucaciones de EQA. Al recoger datos durante un año, se podrían obtener 12 pares dedatos para el análisis de regresión de este estudio.

Además del tamaño muestral, la distribución de valores es otro elemento importante para el análisis de regresión. Por ejemplo, cuando el análisis de regresión se realizó empleando pares de datos que incluían concentraciones de colesterol HDL de entre 46 y 134 mg/dL, la ecuación de regresión resultante mostraba una desviación del 1,46 % y del 11 % para concentraciones de 73,8 y 28,6 mg/dL, respectivamente. Esto se atribuyó a la falta de pares de datos con una baja concentración en el conjunto de datos empleado para el análisis de regresión.

Al igual que ocurre con los valores objetivo de EQA, los valores objetivo de IQC no se asignan empleando métodos de referencia. Además, aunque se recomienda que sea el laboratorio el que defina los valores objetivo de IQC [33], normalmente se emplean los valores medios proporcionados por el fabricante del ensayo en los “*inserts”* de la caja de control. Por lo tanto, la incertidumbre de los valores objetivo complica también el cálculo del sesgo basado en datos de IQC. Por otro lado, el método basado en la media de los valores de desviación de los resultados de IQC se basa en más funciones de datos que aquellos basados en los resultados de EQA, lo que hace que la estimación del sesgo basada en datos de IQC sea más robusta frente a errores aleatorios.

Existe heterogeneidad entre estudios, en lo relativo a la estimación del sesgo. Los estudios que evalúan la calidad analítica de un nuevo sistema mediante métrica Sigma suelen calcular el sesgo a partir de datos obtenidos mediante el estudio comparativo de métodos [7], [8], [9]. Por otro lado, cuando la métrica Sigma se utilizaba para diseñar estudios de IQC o evaluar la calidad analítica de un sistema existente, el sesgo se calculó a partir de resultados de IQC y EQA [10], [11], [12], [13], [14], [15].

Tran et al. [17] evaluó la métrica Sigma de 20 magnitudes químicas en el analizador Beckman Coulter AU680 empleando el método de estimación del sesgo basado en el análisis de regresión de los resultados de EQA. De acuerdo con los resultados de este estudio, los autores calcularon una calidad Sigma <3 para la albúmina, la creatinina, el sodio y el cloro en el nivel 1 de IQC. Los autores también documentaron que el calcio, lasproteínas totales, la glucosa y la CK tenían un valor Sigma de <3 en el nivel 1 de IQC. Además, en 6 de las 8 magnitudes en el nivel 1 de IQC, se han descrito los mismos hallazgos en el nivel 2 de IQC. La diferencia en los valores Sigma entre los dos estudios se atribuyó a los diferentes valores de ETa empleados para las magnitudes, excepto para la glucosa y la CK. Los valores de Sigma inferiores obtenidos para la glucosa y la CK se debieron a los valores de sesgo superiores del citado estudio. La diferencia en los valores de sesgo entre los dos estudios podría explicarse, al menos en parte, a las distintas plataformas analíticas empleadas. Además, la distribución de datos empleados en el análisis de regresión podría haber influido en los valores de sesgo. Desafortunadamente, el citado estudio omite algunos datos relevantes.

En otro estudio, Nar y Emekli 14 calcularon la métrica Sigma de 18 magnitudes de inmunoanálisis en un analizador Cobas e601, 17 de los cuales coincidían con los de nuestro estudio, para evaluar la calidad analítica. Los autores calcularon el sesgo determinando la diferencia entre los valores objetivo (valores medios) en los *inserts* de control y los valores medios observados. Concidiendo con el presente estudio, documentaron un valor Sigma <3 para FT4. Además, a diferencia de este estudio, se informó un valor Sigma <3 para AFP. Por otro lado, obtuvieron un valor Sigma >6 para el ácido fólico, mientras que en nuestro estudio obtuvimos un valor Sigma <3. Cuando los laboratorios emplean la misma plataforma analítica y reactivos, se debería obtener una calidad Sigma similar. Por tanto, en el caso de obtener una baja calidad Sigma, se deberán adoptar medidas en relación a aspectos locales que influyen en la calidad analítica. Entre dichos factores se encuentran el mal funcionamiento de los equipos e instrumentos, aspectos relacionados con los reactivos, el manejo de las muestras, o posibles errores relacionados con el usuario del equipo.

Kumar y Mohan 12 evaluaron la calidad Sigma de 16 magnitudes químicas en un analizador VITROS 4600 empleando el sesgo medio de los valores mensuales de sesgo basados en los resultados de EQA. Los autores documentaron una calidad Sigma similar a la de este estudio para el sodio y la albúmina, aunque los valores Sigma fueron superiores para la creatinina. A pesar de haber obtenido valores de desviación e imprecisión similar para la creatinina, la diferencia observada en los valores Sigma se debieron a la diferencia en los valores ETa utilizados en los estudios. Los autores seleccionaron un ETa del 15 % basándose en el estado del arte, mientras que en nuestro estudio se empleó un ETa del 7,5 % basado en la variabilidad biológica. También se han descrito valores Sigma <3 para la urea empleando un ETa del 9 % basado en el estado del arte, mientras que en nuestro estudio se obtuvo un valor Sigma >6 empleando un ETa del 17,6 % basado en su variabilidad biológica.

La influencia de esta heterogeneidad en el cálculo de la desviación sobre la métrica Sigma se ha analizado en diversos estudios [10, 15]. En un estudio llevado a cabo por Guo et al. [10], la métrica Sigma para 10 magnitudes químicas se estimó aplicando dos métodos para calcular la desviación y la imprecisión, uno de ellos basado únicamente en los resultados de IQC, mientras que el otro solo se basó en los resultados de EQA. En el método basado en los resultados IQC, la desviación se calculó mediante comparación con el grupo par del programa de IQC. Cabe mencionar que, en el método basado en los resultados EQAC, los autores emplearon muestras de EQA para estimar la imprecisión tras realizar un estudio de precisión durante un periodo relativamente corto, y estimar el sesgo. En consonancia con los resultados del presente estudio, los autores obtuvieron niveles Sigma significativamente diferentes con los dos métodos, lo que podría afectar a los resultados de la selección de reglas de IQC.

En otro estudio más reciente [15], Wauthier et al. compararon la métrica sigma de 20 magnitudes, calculada utilizando dos métodos de cálculo de sesgo basados en la comparación de grupos par del programa EQA o IQC. A diferencia de los hallazgos del presente estudio, los autores concluyeron que la fuente de sesgo fue menos decisiva para la métrica Sigma. Al evaluar el impacto del método de cálculo de sesgo sobre la métrica Sigma, los autores clasificaron la métrica Sigma como <3, 3–6, y >6. Dicho de otro modo, no se evaluaron las diferencias en la métrica Sigma de entre 3 y 6, que son importantes a la hora de seleccionar las normas de IQC y la frecuencia de las mediciones. Esto podría dificultar la identificación del efecto de los métodos de estimación del sesgo sobre la métrica Sigma resultante.

Además, junto al método convencional, Coskun et al. introdujeron un nuevo modelo para calcular la métrica Sigma en medicina de laboratorio [34]. En este nuevo modelo, la métrica Sigma se determina incorporando la imprecisión analítica y los límites de tolerancia. A diferencia del método tradicional, el sesgo no se incluye directamente en la ecuación de la métrica Sigma. En su lugar, el valor de la métrica Sigma se convierte en defectos por millón de oportunidades, presumiendo la presencia de un sesgo de 1,5 de desviación estándar. Por consiguiente, el modelo propuesto facilita la evaluación de métodos analíticos empleando la métrica Sigma. Sin embargo, este modelo considera únicamente los límites de tolerancia basados en la variabilidad biológica, independientemente de los métodos empleados para definir los límites de tolerancia. Esta limitación podría restringir la aplicación del nuevo modelo a los procesos analíticos de laboratorio, ya que no se dispone de datos de variabilidad biológica para todas las magnitudes, o la variabilidad puede no ser relevante para algunas de ellas. Así mismo, cabe mencionar que este modelo no aporta una metodología para la selección o el diseño de reglas de IQC [35]. Por lo tanto, en el presente estudio únicamente se empleó la fórmula tradicional para calcular la métrica Sigma.

Este estudio tiene algunas limitaciones. En primer lugar, lo ideal sería que el sesgo se calculara mediante comparación con los métodos de referencia [16], sin embargo, en nuestro estudio, el sesgo se calculó a partir de datos de IQC y EQA. De este modo, los valores de sesgo aportados deberían ser considerados relativos, más que absolutos. La selección de ETa objetivo de múltiples fuentes supone también una limitación. Si se eligiera un valor de ETa diferente del empleado en nuestro estudio, se podrían obtener diferentes métricas Sigma. Por desgracia, no se dispone de valores de ETa universalmente aceptados para calcular valores Sigma. El Grupo de Trabajo de la IFCC para la estandarización de la HbA_1c_ recomienda un valor de ETa específico para calcular la métrica Sigma de HbA_1c_ [22]. Dicho grupo de trabajo, ha definido el valor de ETa teniendo en cuenta la calidad analítica de los métodos existentes actualmente. Como la HbA_1c_, establecer valores ETa específicos para calcular los valores Sigma podría ayudar a resolver el problema de decidir sobre la fuente apropiada de ETa.

En conclusión, la métrica Sigma podría diferir dependiendo de si el sesgo procede del cálculo de la media o del análisis de regresión de los resultados de EQA o si se obtiene a partir de los resultados de IQC. Esto se debe tener en cuenta a la hora de evaluar la calidad analítica o diseñar eventos de IQC aplicando el método Seis Sigma.

## Supplementary Material

Supplementary Material

## Material Suplementario

Este artículo ofrece material suplementario (https://doi.org/10.1515/almed-2023-0095).
