# Supplementary material for: Comparación de la métrica Sigma calculada con tres métodos de estimación del sesgo en 33 magnitudes químicas y 26 de inmunoensayo
Source: Adv Lab Med. 2023 Aug 28;4(3):246–57. [Article in Spanish] doi: 10.1515/almed-2023-0095 (PMC10756148; doi:10.1515/almed-2023-0095)
Supplement: Supplementary file 1 — Supplementary Material [file j_almed-2023-0095_supp_001.docx]

**Tabla suplementaria 1**: Valores de sesgo mensuales obtenidos de los programas de EQA

| Magnitud | Valores de sesgo (%) | | | | | | | | | | | |
| --- | --- | --- | --- | --- | --- | --- | --- | --- | --- | --- | --- | --- |
|  | Marzo  2019 | Abril  2019 | Mayo  2019 | Junio  2019 | Julio  2019 | Agosto  2019 | Septiembre  2019 | Octubre  2019 | Noviembre 2019 | Diciembre  2019 | Enero  2020 | Febrero  2020 |
| Albúmina | 1,6 | 9,4 | 1,9 | 1,7 | 1,4 | -2,2 | 4,4 | 12,2 | -0,6 | -5,4 | 4,4 | -2,7 |
| ALP | -0,4 | 10,3 | 1,3 | 1,8 | 0,7 | -6,4 | -1,1 | -0,1 | -0,1 | -1,5 | 1,9 | 0,2 |
| ALT | 0,7 | -1,7 | 0 | -0,3 | 1,7 | -1,4 | 3,6 | 0,5 | 2,1 | -2,6 | 2,3 | 5,5 |
| Amilasa | -0,2 | -5,4 | 1,5 | 2,1 | 0,2 | -6,6 | 0,6 | -1,8 | -1,8 | -2,7 | 0,4 | -6,6 |
| Antiestreptolisina O | 0,9 | -2 | -9,1 | -2,3 | 7,2 | 4 | * | 1,3 | 4 | 3 | 6 | 2,9 |
| AST | 0,5 | 5,5 | 3,8 | 5,6 | 3 | -5,7 | 3,5 | 2 | 0,9 | -3,3 | 4,5 | 10,7 |
| Bilirrubina, directa | -3,2 | 4,3 | 0,9 | -1,1 | -1,8 | -3,9 | 1 | -0,8 | -0,5 | -1,8 | -0,4 | -1,7 |
| Bilirrubina, total | -2,2 | -1,6 | 1,1 | -1,3 | -0,6 | -2,7 | 2,2 | -0,2 | 2,8 | -3,4 | 1,5 | 3 |
| Proteína C-reactiva | 1,6 | -4,1 | 0,2 | 3,3 | 2,4 | 3,5 | * | 2,8 | 0,2 | 1,7 | -2,4 | 2,3 |
| Calcio | 2,6 | 1,7 | 2,1 | 0,9 | 1,4 | -6 | -3 | -0,7 | 1,6 | -4,4 | -1 | 0,2 |
| Cloro | 0,2 | -1,3 | -2 | -3 | 0,3 | -6,5 | 0,7 | -1 | -3,2 | 2 | 0,6 | 3,2 |
| Colesterol, HDL | -4,4 | -2,3 | -0,2 | 0 | -4,8 | -8,4 | -2,3 | 6,7 | 4,7 | -4,1 | 2,8 | 1,5 |
| Colesterol, LDL | 7 | 6,7 | 5,2 | * | 0 | -3,1 | 2,6 | 0,9 | 2,3 | 3,8 | -0,7 | -2,3 |
| Colesterol, total | 1,7 | 2,5 | 4,5 | 4,5 | 3,6 | -3,8 | 0,4 | 0,7 | 0,5 | -3 | -0,5 | 1,5 |
| Creatina quinasa | -1 | -1,1 | -2,4 | -2,4 | 0,2 | -3,4 | 2,3 | 0,4 | -1,9 | -2,2 | 3,3 | 1,7 |
| Creatinina | 0,2 | -3,1 | 4,1 | 5,1 | 2,6 | -0,9 | 0,7 | 4,1 | 0,8 | -9,6 | 1 | 1,7 |
| D-dímero | -3,3 | 8,79 | 11,7 | 14,4 | 3,4 | -1,5 | -5 | -3,7 | 5,8 | 3,1 | 2,3 | 4 |
| GGT | 4,5 | 8,2 | 3,1 | 5 | 1,5 | -3,6 | 1,5 | 1 | 3,2 | -0,9 | 1,7 | 6 |
| Glucosa | 1,4 | 2,2 | 1,6 | -0,1 | -0,5 | -5,6 | 0,3 | -0,2 | 0,7 | -3 | -0,2 | 2,3 |
| Hemoglobina A1c | -0,2 | 2,8 | 2,3 | -0,6 | 3,7 | -8 | 0,2 | -0,1 | 0,7 | 0,7 | -1,1 | 1,8 |
| Hierro | -1,1 | -0,9 | -0,1 | 1,9 | -2 | -0,3 | 4,5 | 2,5 | 0,7 | -3 | 1,5 | -5,1 |
| LDH | -0,8 | 2,1 | 1,8 | 2,7 | 0,1 | -4,7 | -0,2 | 0,4 | 1,4 | -4,1 | 0 | 1,9 |
| Lipasa | -1,7 | -10,4 | 1,6 | -1,1 | -2,4 | -3,2 | -1,1 | -3,5 | 2,9 | -1,5 | -2,7 | 0 |
| Litio | 5,7 | 0,6 | -1,6 | 2,6 | -1,1 | -0,4 | 2,9 | 2,4 | -11,6 | -1,4 | 1,6 | 6,3 |
| Magnesio | 0,1 | 2,1 | 2,9 | 2,6 | 2,3 | -2,9 | -0,4 | 2,2 | 0,8 | -4,7 | 0,6 | 3,6 |
| Fósforo | 0,6 | -3,2 | -2,5 | 0,1 | -0,1 | -2,4 | 1 | -0,3 | 2,6 | -1,8 | 3,9 | -3,6 |
| Potasio | -0,4 | -1,4 | 0,8 | 0,6 | 1,9 | -3,4 | 1,5 | -1,1 | -0,4 | 0 | 1,1 | 3,8 |
| Proteínas totales | -0,4 | -1,6 | 5,2 | 0,4 | 2 | -7,4 | 2,4 | 1,4 | 2,4 | -1,3 | 3,7 | 5,3 |
| Factor reumatoide | 2,7 | -1,5 | -4,8 | 1,1 | 1,4 | -3,8 | * | -0,8 | 0,2 | -1,3 | -2,4 | 0,2 |
| Sodio | 0,6 | 1,4 | 2,2 | 0,6 | 1,5 | -3,1 | 1,6 | -0,3 | -0,7 | -0,1 | 1,7 | 3,7 |
| Triglicéridos | -0,4 | 2,7 | 1,3 | 3,5 | 0,4 | -6,6 | -1,7 | 3,1 | 5,2 | -2,8 | 1,9 | 2,5 |
| Urea | 4,7 | 5,4 | 3,9 | 1,7 | -0,6 | -1,2 | 2,5 | -1,2 | -1,3 | -4,5 | -1,5 | -3,2 |
| Ácido úrico | -2,6 | 1 | 3,8 | 2 | 0,9 | -5,2 | -0,5 | -2,6 | -3,8 | -3,1 | -0,2 | 1,3 |
| 25-OHVitamina D | 23,1 | 15,1 | 15,8 | 21,6 | 2,2 | -4,9 | 10,7 | 8,7 | 21,4 | 9,7 | 4,9 | 4,6 |
| AFP | 7,1 | 8,8 | 7,3 | 9,5 | 11,8 | 1,4 | 6,4 | 10,5 | 1,7 | 1,1 | 8,4 | 2,2 |
| CA 125 | 8,9 | 13,9 | 7,7 | 9,6 | 8,8 | 0,6 | 6,4 | 11 | 10,1 | 0,7 | 13,3 | -7 |
| CA 15-3 | 10,2 | 9,2 | 6,2 | 5,3 | 14 | 0,2 | 3,8 | 6,3 | 3,5 | -0,1 | 3,8 | -11 |
| CA 19-9 | 0,9 | 9,1 | 2,6 | 4,4 | 8,1 | 2,1 | 7,2 | 6,9 | 7,5 | 10,3 | 17 | 10,8 |
| CEA | 6,6 | 7 | 6,3 | 5 | 5,6 | -2,5 | 2,4 | 4 | 2,1 | -1,8 | 8,2 | 0,3 |
| Ferritina | 7,6 | 15,5 | 1,5 | 10,7 | 10,5 | -7,4 | 2,8 | 11,5 | -5,4 | 2,1 | 2,5 | 3,5 |
| Folato | 6,2 | 2,8 | 4,1 | -2,1 | 3,5 | 10,5 | -9,3 | -1,2 | 25,1 | 9,1 | 10,3 | 17,2 |
| T3 libre | 3 | 9,8 | 2,5 | 5 | -0,1 | 1,1 | 4,8 | 6,5 | 5,3 | 0,3 | 3,9 | -8,8 |
| T4 libre | -0,1 | 5 | 5 | 4,2 | -3,3 | -3,8 | 1,4 | 2,8 | 1,8 | -0,6 | 5,3 | -0,4 |
| FSH | 7,3 | 11 | 10,2 | 10,5 | 9,7 | 3,9 | 5,6 | 5,4 | 2,3 | 2,7 | 5,8 | -1,3 |
| hCG | 17 | 8,2 | 10,1 | 5,6 | 4,5 | 13,6 | 8,6 | 4 | 0,1 | 5,2 | 3,2 | 3,5 |
| IgE | 1,4 | 8,5 | 11 | 6,5 | 8,6 | 1,2 | 8,6 | 8,6 | 8,1 | 4,7 | 3,6 | 4,4 |
| Insulina | 6,4 | 3,7 | 5,3 | 1,5 | 6,5 | -1,6 | 4,9 | 5,1 | -2,5 | 2,9 | 6,5 | 2,7 |
| LH | 7,5 | 5,6 | 1,6 | 4,5 | 3,9 | -5,2 | 0,5 | 3,7 | -4,6 | -1,6 | 0,5 | 6,3 |
| NT-ProBNP | -2,4 | 19,3 | 3,9 | -0,3 | 3,08 | 4,8 | 2,3 | 0,5 | -2,2 | -0,38 | -3 | 2,7 |
| Estradiol | -1 | 2,9 | -0,3 | 2,3 | -0,6 | 16,6 | 5,3 | 7,5 | 6,9 | -0,8 | 3 | 3,7 |
| PTH | -4,7 | 8 | -17,4 | -15 | 10,9 | -1,9 | 14 | 5,8 | -16,6 | 0,1 | -0,1 | 4 |
| Procalcitonina | 4,5 | -0,2 | 5,3 | 6,1 | 3,4 | 3 | 5,3 | 5 | 2,2 | -1,5 | 2,3 | 2,6 |
| Prolactina | 8,1 | 8,4 | 9,6 | 5,9 | 6,5 | 2,4 | 1,8 | 2 | 1,7 | 0,3 | 4,9 | 2,9 |
| PSA, libre | 1,8 | 3,7 | 5,6 | 5,2 | 3,2 | -0,6 | 4,4 | 1,6 | 7,2 | 1 | 3,6 | 6,9 |
| PSA, Total | 8,5 | 4,4 | 10 | 8 | 8,7 | 1,8 | 5,4 | 7,9 | 6,2 | 4,7 | 1,5 | 3,6 |
| Testosterona | -5,1 | 10,1 | -0,1 |  | 0,5 | 0,3 | 4 | 8,4 | 12,4 | 2,1 | 2,7 | 2,5 |
| Troponina Ths | -3,1 | -9,9 | -6,5 | 3,2 | -15,5 | 1,8 | -5,1 | 7,9 | 6,8 | -9,81 | 6 | 6,2 |
| TSH | 1,2 | 2,9 | 1,6 | 2,4 | 1,5 | 0,3 | 2,3 | 0 | -1,2 | -1 | 5,9 | 2,7 |
| Vitamina B12 | 5,6 | 6,5 | 6,2 | 6,9 | 0,8 | -2,5 | -2,6 | -3 | 10,3 | -1,6 | 3,1 | 5,3 |

*No existen datos disponibles para el estudio EQA correspondiente

**Tabla suplementaria 2:** Valores de sesgo mensuales empleando los resultados del nivel 1 de IQC

| Magnitud | Valor objetivo  (Nivel 2 del IQC) | Valores de % desviación | | | | | | | | | | | |
| --- | --- | --- | --- | --- | --- | --- | --- | --- | --- | --- | --- | --- | --- |
|  |  | Marzo  2019 | Abril  2019 | Mayo  2019 | Junio  2019 | Julio  2019 | Agosto  2019 | Septiembre  2019 | Octubre  2019 | Noviembre 2019 | Diciembre  2019 | Enero  2020 | Febrero  2020 |
| Albúmina | 32,7 | -0,23 | -0,36 | 0,3 | 0,49 | 2,6 | -2,2 | 1,53 | 4,62 | 1,36 | -1,14 | 1,49 | -0,07 |
| ALP | 94,9 | -1,1 | 0,47 | -0,02 | 1,35 | -5,48 | -5,27 | -4,14 | -3,03 | -1,38 | -3,32 | -3,99 | -5,97 |
| ALT | 48 | -3,87 | -2,56 | -2,98 | -1,75 | 0,87 | -1,98 | -1,3 | -2,22 | -1,75 | -1,94 | 1,58 | 2,58 |
| Amilasa | 76,3 | -1,22 | -0,22 | -0,39 | 0,61 | -1,11 | -1,1 | -2,09 | -1,43 | -1,09 | -0,49 | -2,12 | -2,02 |
| Antiestreptolisina O | 122 | 2,21 | -1,3 | -1,85 | -0,82 | 4,66 | 5,05 | 8,49 | 4,89 | 5,25 | 5,82 | 1,14 | 0,2 |
| AST | 46,4 | -2,07 | 0,98 | 0,71 | 0,79 | 1,83 | -0,66 | -0,15 | 0,08 | 0,21 | -0,87 | 0,88 | 3,45 |
| Bilirrubina, directa | 0,994 | -1,95 | -1,25 | -2,05 | -1,49 | -2,14 | -3,26 | -2,99 | -2,53 | -2,17 | -2,76 | -2,03 | -1,38 |
| Bilirrubina, total | 1,05 | -2,03 | -1,55 | -2,94 | 0,14 | 2,27 | 0,06 | 1,66 | 1,18 | 1,96 | 1,38 | 3,34 | 2,96 |
| Proteína C-reactiva | 7,96 | -0,47 | -0,88 | 2,56 | 6,89 | 2,9 | 3,64 | 2,75 | 3,69 | 2,67 | 6,06 | 3,6 | 1,87 |
| Calcio | 8,9 | 1,68 | 0,89 | -0,84 | 0,13 | 0,43 | -2,52 | -2,84 | -1,31 | 0,24 | 1,45 | -0,03 | -0,32 |
| Cloro | 76,5 | 4,07 | 1,12 | -0,48 | -0,04 | 8,76 | -3,55 | -1,92 | 0,16 | -0,45 | 1,14 | -3,02 | -1,46 |
| Colesterol, HDL | 28,6 | -3,43 | -5,69 | -0,18 | 0,57 | -0,35 | -3,67 | -2,95 | 3,99 | 4,85 | 5,31 | 2,6 | 1,53 |
| Colesterol, LDL | 56,1 | 1,69 | 2,67 | 8,19 | 9,51 | 2,45 | -2,32 | 2,02 | 1,93 | 2,96 | 3,75 | -3,13 | -3,82 |
| Colesterol, total | 90,9 | 0,48 | 1,48 | 3,17 | 4,22 | 4,08 | 2,58 | 3,27 | 1,99 | 2,94 | 8,29 | 1,42 | 0,93 |
| Creatina quinasa | 155 | -0,22 | -0,73 | -0,36 | 0,19 | 0,44 | -0,15 | 0,46 | 1,31 | 0,51 | -0,98 | -0,61 | -1,54 |
| Creatinina | 1,07 | 2,1 | 2,14 | 4,66 | 4,65 | -0,02 | 0,29 | 1,75 | 4,13 | 2,88 | 3 | 2,55 | -0,56 |
| D-dímero | 0,82 | -1,59 | -1,71 | -2,83 | -7,97 | -5,48 | -6,63 | -5,46 | -5,03 | 0,88 | 0,65 | -2,51 | -3,37 |
| GGT | 53,3 | 1,63 | 1,92 | 2,1 | 3,08 | 2,5 | 1,5 | 0,36 | 0,7 | 1,54 | -5,46 | -0,16 | -1,58 |
| Glucosa | 100 | 2,41 | 2,66 | 2 | 2,32 | 1,46 | 0,68 | 1,16 | 1,65 | 2,82 | 1,77 | 2,05 | 1,26 |
| Hemoglobina A1c | 5,7 | -0,6 | -0,94 | 0,24 | -0,96 | -2,86 | -0,76 | -0,8 | -1,25 | -0,98 | -0,54 | -0,13 | 0,49 |
| Hierro | 105 | 1,44 | 1,02 | 1,81 | 2,74 | 1,33 | 2,41 | 2,75 | 0,15 | 2,02 | 1,96 | 1,2 | -0,07 |
| LDH | 170 | -0,41 | 1,47 | 1,66 | 2,44 | 2,5 | 2,92 | 3,57 | 3,84 | 3,92 | 1,94 | 3,8 | 2,5 |
| Lipasa | 45,6 | -0,75 | -1,54 | -1,6 | -1,85 | -2,18 | -0,45 | -0,46 | -1,32 | 1,05 | -6,51 | -0,37 | -0,49 |
| Litio | 0,9 | 6,3 | 3,89 | 3,06 | 5,74 | 0,88 | 2,92 | 3,2 | 0,91 | 1,13 | 5,52 | 5,37 | 5,2 |
| Magnesio | 1,98 | -1,65 | -1,85 | -1,67 | -0,68 | -0,08 | 0,55 | -0,84 | -0,85 | 0,32 | 2,26 | -1,77 | -1,09 |
| Fósforo | 4,15 | -2,03 | -2,03 | -2,05 | 0,32 | 1,28 | 1,93 | 2,29 | 1,61 | 3,03 | 1,94 | 2,68 | 2,39 |
| Potasio | 3,66 | 0,89 | 1,2 | 0,63 | 1,78 | 0,14 | 0,84 | 0,67 | 0,36 | 0,34 | -0,52 | 0,22 | -0,17 |
| Proteínas totales | 49,3 | -1,18 | -1,21 | 0,96 | 2,36 | 1,27 | -1,39 | 0,98 | 1,97 | 2,89 | 2,51 | 2,25 | 2,47 |
| Factor reumatoide | 20,5 | 1,9 | 4,04 | 4,59 | 4,48 | 2,18 | 0,51 | -0,61 | 2,52 | 4,02 |  | -0,22 | 1,78 |
| Sodio | 113 | -0,1 | 0 | -0,28 | 0,19 | 0,8 | 1,26 | 1,29 | 1,01 | 1,51 | -0,07 | 0,25 | -0,45 |
| Triglicéridos | 121 | 0,38 | 0,95 | 0,97 | 2,8 | 2,9 | -1,1 | -0,79 | 2,22 | 2,86 | 1,14 | 1,42 | 1,41 |
| Urea | 39,3 | 2,3 | 1 | -0,28 | 0,27 | 1,73 | 3,41 | 2,73 | 2,57 | 1,61 | -0,23 | -1,87 | -3,15 |
| Ácido úrico | 4,6 | -0,56 | -0,35 | 2,03 | 3,76 | 2,03 | 0,74 | 0,02 | -2,08 | -1,37 | 0,37 | -0,93 | -1,34 |
| 25-OHVitamina D | 12,92 | -10,36 | -12,45 | -3,24 | -1,28 | -5,01 | -7,83 | -6,86 | -8,92 | -3,12 | -1,48 | -2,07 | 0,45 |
| AFP | 12 | 0,87 | 2,05 | 2 | 3,83 | 3,9 | 2,2 | 3,6 | 1,05 | -3,42 | -1,15 | -0,39 | 2,02 |
| CA 125 | 33,46 | 6 | 6,31 | -3,07 | -3,88 | -4,82 | -6,95 | -5,99 | -5 | -2,42 | -7,2 | -2,48 | -0,9 |
| CA 15-3 | 20,81 | 1,5 | -3,34 | -9,02 | -9,27 | -7,2 | -9,54 | -12,51 | 0,55 | -0,04 | 2,58 | -2,66 | 2,93 |
| CA 19-9 | 25,05 | 0,89 | 0,95 | -2,03 | 1,29 | 5,91 | 10,87 | 11,88 | 2,87 | 3,28 | 3,36 | 1,11 | 1,35 |
| CEA | 5,17 | -0,58 | -0,51 | -1,7 | -0,91 | -0,21 | -1,59 | 2,68 | 1,05 | -1,15 | -4,55 | -2,67 | -3,24 |
| Ferritina | 25,37 | -0,91 | -1,04 | -6,17 | 0,81 | 0,58 | -6,08 | -5,71 | -3,34 | -4,84 | -7,84 | -0,47 | 1,26 |
| Folato | 4,598 | 3,7 | 15,69 | 4,18 | 12,85 | 14,45 | 7,81 | -0,14 | 3,62 | 7,82 | 6,72 | -5,22 | 1,07 |
| T3 libre | 5,5 | -2,57 | 4,55 | 2,51 | 4,8 | 2,99 | 4,03 | -0,95 | 1,11 | 3,12 | 1,82 | 0,63 | 2,53 |
| T4 libre | 1,18 | -7,45 | -2,86 | -0,75 | -0,92 | -3,13 | -1,71 | -2,18 | -0,13 | -0,56 | 3,02 | 3,06 | -2,52 |
| FSH | 18,51 | 1,43 | 4,28 | 1,18 | 1,22 | 1,56 | -0,26 | -2,06 | -2,8 | -4,48 | -3,33 | 2,46 | -3,77 |
| hCG | 5,11 | 6 | 2,97 | -0,61 | 0,6 | -1,29 | 5,83 | -3,89 | -7,43 | -7,48 | -4,25 | -4,76 | 1,68 |
| IgE | 123,78 | -10,52 | 0,23 | 0,74 | 1,01 | 0,07 | -0,68 | 5,26 | 3,46 | 5,6 | 4,54 | 6,36 | -1,76 |
| Insulina | 24,46 | 2,14 | -1,46 | -3,8 | -4,01 | -2,13 | -0,11 | -0,4 | -4,11 | -3,21 | -1,27 | 2,03 | 0,17 |
| LH | 9,77 | -2,75 | 1,53 | -0,18 | 0,46 | -0,35 | -4,72 | 3,35 | 3,92 | 3,14 | 3,17 | 6,99 | 1,85 |
| NT-ProBNP | 134 | 5,87 | 1,68 | -2,41 | -0,93 | 5,57 | 4,4 | 4,42 | -1,42 | -0,03 | -2,18 | -2,3 | 0,89 |
| Estradiol | 105 | -11,82 | -5,11 | -9,31 | -11,81 | -9,72 | -4,02 | -4,57 | -1,35 | -1,68 | -4,94 | -10,2 | -1,34 |
| PTH | 51,6 | 7,32 | 6,74 | 7,45 | 5,09 | 7,71 | 10,11 | 9 | 6,42 | 5,31 | -13,58 | 1,62 | 0,77 |
| Procalcitonina | 0,47 | 4,35 | -0,27 | -1,6 | 0,91 | 1,31 | -2,74 | -5,9 | -7,95 | -1,5 | 1 | 2,52 | 6,15 |
| Prolactina | 223,24 | -1,5 | 0,33 | 0,07 | 0,48 | -0,67 | -2,85 | -2,42 | -2,48 | -3,11 | -0,61 | 2,41 | -1,22 |
| PSA, libre | 0,85 | -5,12 | -4,24 | -0,62 | 0,09 | -1,84 | -2,04 | -0,1 | -0,5 | -1,07 | -8,49 | 2,88 | 1,76 |
| PSA, Total | 3,93 | -2,6 | -2,58 | 2,51 | 0,47 | -2,27 | -2,27 | -1,22 | -0,52 | 0,91 | -3,88 | -3,58 | -3,05 |
| Testosterona | 5,8 | -14,04 | 4,36 | 5,97 | -1,5 | 0,44 | 0,7 | -0,55 | 4,68 | 6,59 | 5,24 | -2,9 | -4,15 |
| Troponina Ths | 28,35 | 3,1 | 3,02 | 5,14 | 5,25 | 4,31 | 1,61 | 0,55 | 1,49 | 0,41 | 2,71 | 4,57 | 2,97 |
| TSH | 1,38 | -9,72 | 2,33 | 0,96 | 3,17 | 2,36 | 4,44 | -2,32 | -3,8 | -1,33 | -2,8 | 2,17 | -0,5 |
| Vitamina B12 | 512,01 | -3,62 | 3,9 | -1,65 | 0,85 | 1,56 | 2,01 | -3,09 | -3,56 | -4,26 | -4,51 | -0,87 | 3,18 |

**Tabla suplementaria 3**: Valores de sesgo mensuales empleando los resultados del nivel 2 del IQC

| Magnitud | Valor objetivo (Nivel 2 del IQC) | Valores de %desviación | | | | | | | | | | | |
| --- | --- | --- | --- | --- | --- | --- | --- | --- | --- | --- | --- | --- | --- |
|  |  | Marzo  2019 | Abril  2019 | Mayo  2019 | Junio  2019 | Julio  2019 | Agosto  2019 | Septiembre  2019 | Octubre  2019 | Noviembre 2019 | Diciembre  2019 | Enero  2020 | Febrero  2020 |
|  |  | 3 | 4 | 5 | 6 | 7 | 8 | 9 | 10 | 11 | 12 | 1 | 2 |
| Albúmina | 49 | -1,32 | -0,24 | -0,56 | 0,49 | 0,34 | 1,18 | 1,7 | 4,13 | 0,59 | -1,16 | -0,28 | -2,99 |
| ALP | 234 | -4,65 | -3,66 | -4,21 | -3,41 | -3,28 | 12,22 | 6,69 | 6,25 | 6,24 | 5,92 | 4,66 | -12,39 |
| ALT | 114 | -1,84 | -0,8 | -0,84 | 0,28 | 0,44 | -0,11 | -2,22 | -2,22 | -2,16 | -3,26 | -2,27 | -2,38 |
| Amilasa | 190 | -1,49 | -0,29 | -0,54 | 0,19 | -1,39 | -4,24 | -2,78 | -2,07 | -1,61 | -1,69 | -1,74 | 2,7 |
| Antiestreptolisina O | 257 | -1,45 | -2,28 | -3,33 | -1,12 | 4,96 | 6,69 | 3,73 | 1,32 | 1,36 | 2,82 | 2,26 | 1,33 |
| AST | 136 | 0,03 | 2,01 | 1,52 | 2,11 | 2,57 | 1,58 | 0,62 | 1,37 | 1,39 | 0 | 1,44 | 9,09 |
| Bilirrubina, directa | 2,51 | -1,41 | -0,48 | -1,12 | -0,53 | -1,16 | 0,35 | -2,12 | -1,8 | -1,84 | -2,2 | -1,2 | -0,07 |
| Bilirrubina, total | 3,83 | -0,11 | 0,07 | -0,72 | 1,31 | 1,77 | 3,37 | 1,46 | 2,2 | 3,18 | 2,46 | 2,47 | 2,84 |
| Proteína C-reactiva | 41,9 | -7,64 | -7,33 | -3,2 | 0,91 | -0,49 | 29,89 | -2,94 | -1,95 | -1,43 | -0,6 | -0,22 | -0,12 |
| Calcio | 13,6 | 1,7 | 1,33 | -0,53 | 0,63 | 0,1 | -3,04 | -2,97 | -1,33 | 0,25 | 1,03 | -0,05 | 1,38 |
| Cloro | 105,2 | 1,97 | 0,13 | -1,46 | -1,13 | -1,21 | -1,42 | -4,51 | -2,47 | -3,27 | -2,02 | -2,1 | -0,61 |
| Colesterol, HDL | 73,8 | -0,09 | -0,01 | 4,6 | 4,79 | 3,26 | -12,72 | -3,37 | 3,2 | 3,94 | 0,64 | 1,44 | 11,31 |
| Colesterol, LDL | 97,8 | -1,65 | 0,02 | 4,74 | 6,66 | -0,2 | -0,39 | -0,63 | -0,09 | 0,53 | -3,08 | -5,67 | -10,83 |
| Colesterol, total | 171 | -1,42 | 0,64 | 1,01 | 1,65 | 2,34 | -0,8 | 0,4 | -0,04 | 0,87 | 0,17 | -0,93 | -2,68 |
| Creatina quinasa | 268 | -0,8 | -0,46 | -0,99 | -1,17 | -0,1 | -0,43 | 0,32 | 1,03 | 1,04 | -0,31 | -0,56 | -2,14 |
| Creatinina | 4,09 | 0,26 | 1,31 | 2,08 | 2,47 | 0,09 | 2,36 | 1,92 | 3,41 | 3,43 | 2 | 1,37 | -0,67 |
| D-dímero | 3,79 | -1,42 | -1,01 | -1,66 | -0,51 | 1,52 | 1,34 | 1,36 | -0,15 | 1,3 | 2,46 | 3,83 | 2,21 |
| GGT | 241 | 0,6 | 1,68 | 0,32 | 0,44 | 0,53 | 7,08 | -1,34 | -0,8 | 0,31 | -0,02 | -0,83 | -3,32 |
| Glucosa | 232 | 0,23 | 0,94 | -0,04 | 0,27 | -0,21 | 1,44 | -0,6 | 0,18 | 0,89 | 0,43 | 0,54 | -0,4 |
| Hemoglobina A1c | 10,3 | -0,02 | 0,52 | 0,7 | -0,89 | 1,38 | 0,79 | 0,73 | 0,07 | 0,71 | 1,54 | 1,63 | 0,51 |
| Hierro | 239 | 0,08 | 0,85 | 0,59 | 0,93 | 0,21 | 0,86 | 0,82 | -0,98 | 1,1 | 0,89 | 0,59 | -0,21 |
| LDH | 292 | -1,49 | 0,53 | 0,8 | 1,31 | 0,84 | 2,4 | 2,46 | 3,34 | 4,01 | 1,48 | 0,77 | 5,9 |
| Lipasa | 98,7 | -1,61 | -0,84 | -1,3 | -1,85 | -2,59 | -1,07 | -2,43 | -2,46 | 0,04 | -1,45 | -2,51 | 14,78 |
| Litio | 1,73 | 1,04 | 0,96 | 0,91 | 0,87 | -1,39 | 1,73 | -1,14 | -1,03 | -1,27 | 0,46 | 3,62 | 1,49 |
| Magnesio | 3,23 | -2,35 | -1,44 | -2,04 | -0,46 | -0,11 | 1,44 | 0,17 | -0,02 | 0,57 | -1,28 | 0,02 | 0,6 |
| Fósforo | 7,38 | -1,49 | -1,51 | -2,34 | -0,33 | 0,73 | 4,34 | 1,85 | 2,28 | 2,85 | 1,41 | 2,08 | 3,39 |
| Potasio | 6,75 | -0,11 | 0,64 | 0,07 | 0,88 | 0,7 | 3,41 | 0,48 | 0,42 | 0,42 | -0,02 | 0,76 | -0,03 |
| Proteínas totales | 75,6 | -2,38 | -1,79 | 0,31 | 1,69 | 0,05 | 1,7 | 0,28 | 1,11 | 2,12 | 1,51 | 2,26 | 0,86 |
| Factor reumatoide | 51,3 | -0,61 | 1,92 | -2,17 | 1,59 | 0,21 | -2,16 | -2,09 | -0,68 | -0,23 |  | 1,07 | 3,21 |
| Sodio | 135 | 0,2 | 0,81 | 0,46 | 0,76 | 0,55 | 1,1 | 0,36 | 0,46 | 0,88 | 0,11 | 1,15 | 1,36 |
| Triglicéridos | 226 | -2,05 | -1,16 | -1,74 | -0,09 | -0,62 | -6,1 | -2,04 | 0,25 | 0,63 | -0,83 | 0,24 | 6,35 |
| Urea | 117 | 0,92 | -0,31 | -2,19 | -1,71 | -3,03 | 1,57 | -1,15 | -0,8 | -1,15 | -2,79 | -2,89 | -3,78 |
| Ácido úrico | 9,27 | -2,15 | -1,36 | 0,5 | 1,98 | 1,26 | 2,71 | -0,48 | -2,09 | -1,92 | -0,15 | -1,07 | -2,04 |
| 25-OH Vitamina D | 27,63 | -5,4 | -8,48 | -0,99 | 2,83 | -1,96 | -4,37 | -3,51 | -4,67 | -2,34 | 0,99 | -0,66 | 9,02 |
| AFP | 62,8 | -4,3 | -5,41 | -4,54 | -2,39 | -0,02 | -2,8 | -4,17 | -2,52 | -7,36 | -3,27 | -3,18 | -3,39 |
| CA 125 | 95,47 | 3,63 | 3,8 | -0,73 | -2,02 | -2,03 | -5,68 | -5,15 | -3,74 | -1,07 | -4,91 | -1,66 | -1,99 |
| CA 15-3 | 90,88 | -5,09 | -7,3 | -1,32 | -2,98 | 1,92 | -2,87 | -5,24 | -2,19 | -5,89 | 0,25 | 1,03 | 0,17 |
| CA 19-9 | 106,29 | -0,97 | 0,32 | 1,9 | 2,58 | 8,2 | 10,69 | 12,64 | -1,84 | 0,48 | 3,61 | 2,2 | 0,3 |
| CEA | 50,62 | -3,17 | -3,98 | -0,12 | -1,53 | 0,08 | -2,8 | -0,92 | -1,76 | -3,75 | -2,35 | -0,45 | -1,64 |
| Ferritina | 195,6 | -2,84 | -2,94 | -3,39 | 1,99 | 3,52 | -5,07 | -3,98 | 0,81 | -1,63 | 2,11 | -1,59 | -2,74 |
| Folato | 13,3 | 9,34 | 23,52 | 3,95 | 9,79 | 8,85 | 2,27 | -0,57 | 3,22 | 2,85 | -1,16 | 0,99 | 5,86 |
| T3 libre | 25,39 | -11,01 | 1,85 | 1,48 | 3,36 | 1,32 | 5,28 | -0,97 | 0,39 | 1,51 | -0,6 | -5,06 | 1,83 |
| T4 libre | 3,23 | -12,84 | -1,29 | -0,34 | -0,54 | -1,67 | 2,57 | -3,94 | -3,31 | -0,79 | 2,61 | -0,45 | -2,33 |
| FSH | 46,73 | 1,74 | 4,07 | 0,83 | 0,62 | 1,1 | 0,58 | -0,51 | -1,77 | -3,72 | -2,25 | 0,68 | -3,57 |
| hCG | 1106,5 | 0,42 | -0,08 | 0,02 | -1,53 | -2,5 | 1,85 | -2,26 | -4,92 | -5,27 | 18,2 | -2,05 | -2,93 |
| IgE | 287,58 | -9,48 | -1,47 | -0,83 | -1,03 | -0,01 | -1,52 | 1,81 | 4,54 | 7,08 | 6,53 | 3,44 | -1,85 |
| Insulina | 78,24 | -1,1 | -1,58 | -4,15 | -4,21 | -1,96 | 0,16 | 0,41 | -2,74 | -4,27 | -2,1 | -1,68 | 0,45 |
| LH | 48,3 | -1,03 | 0,81 | -0,82 | 0,47 | -1,28 | -4,34 | 3,61 | 1,42 | 1,31 | 0,46 | 0,72 | 2,97 |
| NT-ProBNP | 4550 | 4,55 | -1,17 | -3,29 | -0,53 | 4,6 | 0,87 | 2,1 | -4,12 | -1,69 | -4,16 | -3,97 | -1,26 |
| Estradiol | 556 | -11,25 | -7,39 | -7,09 | -8,13 | -6,88 | -3,69 | -8,36 | -6,86 | -7,98 | -11,25 | -14,69 | -7,76 |
| PTH | 183,55 | 8,67 | 6,95 | 6,54 | 4,39 | 7,82 | 8,15 | 8,7 | 5,24 | 4,88 | -2,66 | 2,94 | 1,97 |
| Procalcitonina | 9,15 | 0,39 | -3,87 | -3,56 | -1,23 | -3,7 | -3,91 | -5,54 | -6,11 | -3,54 | -0,94 | 0,29 | 5,21 |
| Prolactina | 820,44 | -2,09 | 0,01 | 0,19 | 0,71 | -0,96 | -1,66 | -2,31 | -2,4 | -2,86 | -0,5 | -0,4 | -0,6 |
| PSA, libre | 11,47 | -5,74 | -3,61 | 1,39 | 1,28 | 0,28 | -1,7 | 0,49 | -0,15 | -0,26 | 8,48 | 4,78 | 2,52 |
| PSA, Total | 36,88 | -2,88 | -2,35 | 5,34 | 2,01 | 0,48 | -1,4 | 0,12 | 0,53 | 0,79 | -1,13 | -1,98 | -3 |
| Testosterona | 2,51 | -21,88 | -5,73 | -3,63 | -6,77 | -6,32 | -5,04 | -4,62 | 2,85 | 3,67 | 1,27 | -3,92 | -5,04 |
| Troponina Ths | 2122 | 1,95 | 0,91 | 1,21 | 0,78 | 2,51 | -1,15 | -1,39 | -1,05 | 1,09 | 4,27 | 5,65 | 2,96 |
| TSH | 8,18 | -6,82 | 2,32 | 1,04 | 3,14 | 1,8 | 4,72 | 0,68 | -1,38 | -0,21 | -3,29 | 2,71 | 0,12 |
| Vitamina B12 | 958,64 | -1,94 | 4,36 | -0,34 | 0,79 | -0,09 | -1,02 | -4,56 | -0,73 | -4,6 | -3,55 | -0,2 | 4,03 |

**Tabla suplementaria 4:** Análisis de regresión de Passing-Bablok basado en los resultados mensuales del EQA

| Magnitud | Valor objetivo  (Nivel 1 del IQC) | Valor objetivo  (Nivel 2 del IQC) | Ecuación de regresión |
| --- | --- | --- | --- |
| Albúmina (g/L) | 32,7 | 49 | y = -0,18664 + 1,021x |
| ALP (U/L) | 94,9 | 234 | y = -2,06972 + 1,014x |
| ALT (U/L) | 48 | 114 | y = -0,32412 + 1,014x |
| Amilasa (U/L) | 76,3 | 190 | y = -2,01413 + 1,013x |
| Antiestreptolisina O (U/mL) | 122 | 257 | y = 5,29594 + 0,986x |
| AST (U/L) | 46,4 | 136 | y = -0,37633 + 1,035x |
| Bilirrubina, directa (µmol/L) | 17,00 | 42,92 | y = -0,06694 + 0,994x |
| Bilirrubina, total (µmol/L) | 17,96 | 65,49 | y = -0,37060 + 1,004x |
| Proteína C-reactiva (mg/L) | 7,96 | 41,9 | y = 0,67387 + 0,991x |
| Calcio (mmol/L) | 2,22 | 3,39 | y = -0,03946 + 1,027x |
| Cloro (mmol/L) | 76,5 | 105,2 | y = -4,10378 + 1,039x |
| Colesterol, HDL (mmol/L) | 0,74 | 1,91 | y = -0,12435 + 1,051x |
| Colesterol, LDL (mmol/L) | 1,45 | 2,53 | y = 0,01409 + 0,994x |
| Colesterol, Total (mmol/L) | 2,35 | 4,42 | y = -0,00134 + 1,010x |
| Creatina quinasa (U/L) | 155 | 268 | y = -6,77097 + 1,018x |
| Creatinina (µmol/L) | 94,61 | 361,64 | y = -5,74375 + 1,050x |
| D-Dimero (µg/L) | 820 | 3790 | y = -22,35740 + 1,093x |
| GGT (U/L) | 53,3 | 241 | y = 0,28239 + 1,015x |
| Glucosa (mmol/L) | 5,55 | 12,88 | y = -0,05839 + 1,011x |
| Hemoglobina A1c (%) | 5,7 | 10,3 | y = -0,21191 + 1,027x |
| Hierro (µmol/L) | 18,80 | 42,78 | y = -0,85120 + 1,038x |
| LDH (U/L) | 170 | 292 | y = 0,50208 + 1,001x |
| Lipasa (U/L) | 45,6 | 98,7 | y = 0,14841 + 0,97892x |
| Litio (mmol/L) | 0,9 | 1,73 | y = 0,01097 + 1,005x |
| Magnesio (mmol/dL) | 0,81 | 1,33 | y = -0,01941 + 1,032x |
| Fósforo (mmol/L) | 1,34 | 2,38 | y = -0,02559 + 1,019x |
| Potasio (mmol/L) | 3,66 | 6,75 | y = -0,07989 + 1,022x |
| Proteínas totales (g/L) | 49,3 | 75,6 | y = -0,56485 + 1,029x |
| Factor reumatoide (U/mL) | 20,5 | 51,3 | y = -0,56148 + 1,009x |
| Sodio (mmol/L) | 113 | 135 | y = -10,33204 + 1,079x |
| Triglicéridos (mmol/L) | 1,37 | 2,55 | y = -0,00027 + 1,014x |
| Urea (mmol/L) | 14,03 | 41,78 | y = -0,31149 + 1,014x |
| Ácido úrico (mmol/L) | 0,27 | 0,55 | y = -0,00690 + 1,012x |
| 25-OH Vitamina D (nmol/L) | 32,25 | 68,96 | y = 2,52773 + 1,068x |
| AFP (kIU/L) | 12 | 62,8 | y = -0,80798 + 1,091x |
| CA 125 (kIU/L) | 33,46 | 95,47 | y = -0,43970 + 1,092x |
| CA 15-3 (kIU/L) | 20,81 | 90,88 | y = -1,43687 + 1,08x |
| CA 19-9 (kIU/L) | 25,05 | 106,29 | y = -2,36323 + 1,108x |
| CEA (ug/L) | 5,17 | 50,62 | y = -0,19772 + 1,053x |
| Ferritina (ug/L) | 25,37 | 195,6 | y = -5,65467 + 1,121x |
| Folato (nmol/L) | 10,42 | 30,14 | y = -0,06045 + 1,060x |
| T3 libre (pmol/L) | 5,5 | 25,39 | y = -0,05642 + 1,04x |
| T4 libre (pmol/L) | 15,19 | 41,58 | y = 0,68498 + 0,994x |
| FSH (IU/L) | 18,51 | 46,73 | y = -0,72578 + 1,091x |
| hCG (IU/L) | 5,11 | 1106,5 | y = 0,10711 + 1,054x |
| IgE (kU/L) | 123,78 | 287,58 | y = -1,35938 + 1,075x |
| Insulina (mU/L) | 24,46 | 78,24 | y = -0,10647 + 1,051x |
| LH (IU/L) | 9,77 | 48,3 | y = -0,86649 + 1,06x |
| NT-ProBNP (ng/L) | 134 | 4550 | y = -1,54916 + 1,03x |
| Estradiol (pmol/L) | 385,46 | 2041,08 | y = 33,59584 + 0,997x |
| PTH (ng/L) | 51,6 | 183,55 | y = -5,26082 + 1,073x |
| Procalcitonina (ug/L) | 0,47 | 9,15 | y = -0,01403 + 1,042x |
| Prolactina (ug/L) | 10,49 | 38,56 | y = 0,05783 + 1,04x |
| PSA, libre (ug/L) | 0,85 | 11,47 | y = 0,01080 + 1,036x |
| PSA, Total (ug/L) | 3,93 | 36,88 | y = 0,17137 + 1,043x |
| Testosterona (nmol/L) | 20,13 | 8,71 | y = -0,03897 + 1,026x |
| Troponina Ths (ng/L) | 28,35 | 2122 | y = -0,99570 + 1,062x |
| TSH (mIU/L) | 1,38 | 8,18 | y = -0,02832 + 1,027x |
| Vitamina B12 (pmol/L) | 377,86 | 707,48 | y = -7,82296 + 1,058x |
